# Exploring the co-occurrence of depression and anxiety symptoms among Chinese vocational high school adolescents through a network approach

**DOI:** 10.3389/fpsyg.2025.1572011

**Published:** 2025-08-11

**Authors:** Yunhan He, Danli Yang, Kaixin Liang, Yunjia Zhang, Hongchuan Zhang, Xinli Chi

**Affiliations:** ^1^School of Psychology, Shenzhen University, Shenzhen, China; ^2^The Shenzhen Humanities and Social Sciences Key Research Bases of the Center for Mental Health, Shenzhen, China; ^3^Department of Psychology, School of Sociology and Psychology, Central University of Finance and Economics, Beijing, China; ^4^Department of Psychology, Faculty of Social Sciences, University of Macau, Macau SAR, China; ^5^Experimental Middle School to Sichuan Institute of Education Sciences, Chengdu, Sichuan, China

**Keywords:** vocational high school students, depression, anxiety, gender differences, network analysis, Bayesian networks

## Abstract

Depression and anxiety, prevalent among adolescents, show higher rates in females than males. However, little is known about symptom-level interactions and gender-specific network characteristics in secondary vocational students. This study adopted network analysis, including Gaussian graph models and directed acyclic graphs, to explore symptom-level relationships between depression and anxiety in secondary vocational students, identifying bridge and core symptoms, potential causation, and gender-specific network characteristics. Involving 7,471 students in a cross-sectional study, we utilized the Patient Health Questionnaire 8-item (PHQ-8) and Generalized Anxiety Disorders 7-item (GAD-7) for symptom measurement. Key findings include: bridge symptom was “sad mood (PHQ2).” Core symptoms were “fatigue (PHQ4),” “loss of control (GAD2),” “trouble relaxing (GAD4),” “sad mood (PHQ2),” “irritability (GAD6),” “excessive worry (GAD3),” and “feeling guilty (PHQ6).” There were numerous cross-disorder links between anxiety and depressive symptoms, predominantly from anxiety symptoms to depressive symptoms. “Irritability (GAD6)” was the initial symptom in the directed network. Females showed higher prevalence of depression and anxiety, and network global strength. This study firstly highlights the complex interplay between depressive and anxiety symptoms in secondary vocational students, with notable gender differences. The insights offer valuable guidance for developing targeted interventions and support strategies for this group.

## 1 Introduction

The largest vocational education system in the world has been built in China, and it starts in vocational high school education. After completing 9 years of compulsory education, students with better academic performance usually attend regular high schools, while those with lower performance attend vocational high schools (Liu et al., 2022). Of note, almost equal numbers of adolescents enter the two education systems. Despite being at the same stage development, vocational high school students often face stigmatization and derogatory labeling regarding their personality traits, behavior patterns, and lower social status (Spruyt et al., 2015). While academic performance differences may partially explain educational tracking, pre-existing mental health challenges could also contribute to lower academic achievement, complicating causal interpretations (Coledam et al., 2022).

Notably, these rates in vocational high school students reached 46.5% for depression and 58.7% for anxiety (Yu et al., 2017), higher than the prevalence of depression and anxiety among regular high school students stands at 28.0% and 26.3% (Yu et al., 2022). Depression and anxiety are not only prevalent but also commonly co-occur, presenting a significant challenge in adolescents’ mental health (Garber and Weersing, 2010). When suffering from either depression and anxiety, the incidence of comorbidity is 10%–75% (Cummings et al., 2014). This comorbidity complicates treatment (Huang et al., 2019), extends disease duration and recovery period (Hung et al., 2020), and leads to more severe outcomes, including higher rates of disability (Zhou et al., 2017). Meanwhile, the relationship between depression and anxiety is intricate and bidirectional, with each potentially predicting the other (Jacobson and Newman, 2017). Therefore, beyond concentrating solely on depression or anxiety, it is important to consider the prevention and intervention strategies for their comorbidity.

A promising framework for investigating depression, anxiety, and their comorbidity is the network approach, which has been widely applied in recent years. Network analysis conceptualizes psychiatric disorders as emerging from dynamic interactions among their constituent symptoms. Within this framework, individual symptoms are represented as nodes in a network, while their statistical associations are depicted as edges (Borsboom and Cramer, 2013). In the current study, this methodology revealed critical structural features of symptom co-occurrence, including the identification of bridge symptoms (e.g., sad mood) that connect distinct symptom clusters, and core symptoms (e.g., fatigue and irritability) that exhibit high centrality within the network. These findings underscore the utility of network analysis in delineating symptom-level mechanisms underlying the comorbidity of depression and anxiety. Network analysis is a promising approach to Fig out important symptoms for targeted interventions. In the context of comorbidity, bridge symptoms and core symptoms are considered important. Symptoms that link disorders are termed “bridge symptoms,” identified by bridge centrality measures, play a critical role in the overlap between disorders (Jones et al., 2021). This insight leads to more targeted and effective interventions, as it highlights pivotal symptoms that drive comorbidity between disorders (Jones et al., 2021). Crucially, bridge symptoms differ across populations and clinical contexts. For instance, in a Gaussian graphical model (GGM) analysis of Chinese adolescents, guilt (PHQ6) was identified as a bridge symptom specifically linking depressive symptoms (e.g., fatigue, sad mood) to anxiety symptoms (e.g., excessive worry, irritability) (Cai et al., 2022). In contrast, anhedonia (PHQ1) emerged as a bridge symptom connecting depressive and generalized anxiety disorder symptoms in Bayesian network analyses of adult populations (Bai et al., 2021; Garabiles et al., 2019). Regarding core symptoms—those with high connectivity to multiple nodes within the network—prior research utilizing undirected network models has demonstrated their utility as ideal therapeutic targets. For example, interventions targeting highly central symptoms (e.g., insomnia, psychomotor agitation) have shown cascading benefits in reducing comorbid symptom severity (Borsboom et al., 2011). However, these studies relied largely on Gaussian Graphical Model (GGM), which lacks the ability to establish causality (Briganti et al., 2023). It remains unclear whether these important symptoms cause the disorder or are a consequence of it.

Bayesian networks structured as directed acyclic graph (DAG) can be used to estimate the activation sequence of symptoms in the network. DAG structures the relationships between variables within a probabilistic framework, reflecting the principles of causal reasoning (Pearl and Mackenzie, 2018; Pearl, 2009). Within a DAG, the joint probability distribution defines the intricate interplay among variables, with each arrow symbolizing a potential causal link. In psychopathology, DAG can elucidate potential causal relationships between symptoms, where each directed edge implies causation from one symptom to another, offering a directional perspective that undirected networks lack (Briganti et al., 2023). For example, DAG analysis has shed light on the causal role of re-experiencing symptoms in the comorbidity of depression and PTSD (Lazarov et al., 2020). However, it appears that this approach has not yet been applied to study the comorbidity of depression and anxiety.

While network analysis has been increasingly applied to identify symptom-level intervention targets in adolescent populations (Fang et al., 2024), no studies to date have employed this methodology to investigate depression and anxiety comorbidity among Chinese vocational high school students. Prior studies have typically employed undirected network models, which provide insights into the associative patterns of symptoms but do not address causality. Moreover, while research has indicated that bridge symptoms and core symptoms differ across populations, there is a lack of study specifically concerning the networks of depression and anxiety among vocational high school students, whether through undirected or directed approach. This study aims to bridge these gaps, This study identifies critical symptoms by employing Gaussian Graphical Models (GGM) and directed acyclic graphs (DAG) to elucidate undirected and directed symptom networks, respectively.

## 2 Materials and methods

### 2.1 Participants and procedures

A cross-sectional study was carried out in Chongqing, China, from 11 March to 17 April 2021, as part of a large mental health screening focusing on depression and anxiety in vocational high school students. Two schools were randomly chosen, and all their first-year students participated. Exclusion criteria were incomplete questionnaire data and failing an attention check (e.g., not selecting the specified option on control questions to filter out inattentive respondents). Of 8,923 submitted questionnaires, 7,471 were valid (83.73%).

The survey utilized “Questionnaire Star,” a popular online tool in China. Students and their guardians were informed about the study’s aim, methods, risks, and benefits before participation. Digital consent was obtained from participants and their guardians, following ethical research standards. The survey took 20–30 min, was conducted in computer classrooms, with instructors facilitating and ensuring access. They were assured that participation was voluntary and anonymous, only those who provided consent and signed the informed consent form proceeded to complete the questionnaire, and data would be kept confidentially and used for research only. The study was approved by the Medical Ethics Committee of Shenzhen University School of Medicine (Granting number: 2020005).

### 2.2 Measures

Depressive symptoms were measured using the Patient Health Questionnaire (PHQ-8), consisting of eight items each representing a depressive symptom. These are rated on a four-point Likert scale from 0 (not at all) to 3 (nearly every day), with the total score indicating depression severity over the past 2 weeks (Kroenke et al., 2009). The reliability and validity of the PHQ-8 has been established in prior research (Kroenke et al., 2009). Although specific validation for the Chinese PHQ-8 is limited, the Chinese version of the PHQ-9, including an additional item assessing suicidal ideation, showed good reliability and validity (Wang et al., 2014). In this study, the PHQ-8 showed excellent internal consistency (Cronbach’s alpha = 0.886) and construct validity, confirmed by confirmatory factor analysis with satisfactory fit indices (χ^2^/df = 27.165, CFI = 0.980, TLI = 0.972, RMSEA = 0.059, SRMR = 0.022).

Anxiety symptoms were measured using the Generalized Anxiety Disorder 7-item Scale (GAD-7). This instrument evaluates seven symptoms of anxiety disorders on a four-point Likert scale, ranging from 0 (not at all) to 3 (nearly every day), with the total score reflecting the severity of anxiety symptoms over the past 2 weeks. The Chinese version has been validated in Chinese adolescent (Sun et al., 2021). In the current study, the GAD-7 also demonstrated excellent internal consistency (Cronbach’s alpha = 0.909) and construct validity, confirmed by confirmatory factor analysis with strong fit indices (χ2/df = 28.122, CFI = 0.982, TLI = 0.970, RMSEA = 0.060, SRMR = 0.021).

### 2.3 Statistic analysis

Descriptive statistics were analyzed using the “bruceR (version 2023.9)” package (Bao, 2023). Our network analysis included a Gaussian Graphical Model (GGM) for undirected networks and a Directed Acyclic Graph (DAG) for directed networks. In GGM, we followed the guideline proposed by Epskamp and Fried (2018a,b), including network estimation and visualization, and calculation of network centrality, network stability and accuracy. Bridge centrality in GGM was measured using Jones et al. (2021) bridge strength method. For the DAG analysis, we adhered to Briganti et al. (2023) tutorial and referenced McNally et al. (2017). Gender differences in network structures were examined using the Network Comparison Test (NCT) developed by van Borkulo et al. (2022).

#### 2.3.1 GGM network

Network estimation and visualization. We modeled symptoms as nodes and their partial correlations as edges using Spearman’s matrices. Considering our large dataset with 35 variables, we chose unregularized models, as recommended by Williams and Rast (2020). We identified the GGM using the ggmModSelect algorithm, which selects the best GGM without regularization, employing a repetitive method and the extended Bayesian information criterion (Williams and Rast, 2020). The analysis was conducted using the R packages “bootnet (version 1.5)” and “qgraph (version1.9.8)” (Epskamp and Fried, 2018b; Epskamp et al., 2012). For network visualization, we applied the Fruchterman and Reingold (1991) algorithm, clustering related nodes and placing less connected ones peripherally.

Node’s bridge centrality and centrality. We used the “networkTools” and “qgraph” R packages to calculate bridge and strength centralities, selecting bridge strength and strength as node attributes (Epskamp et al., 2012; Jones and Jones, 2017). These robust metrics, highlighted in prior studies, quantify node importance (Bringmann et al., 2019; Opsahl et al., 2010). Bridge strength centrality sums absolute edge weights linking nodes across symptom clusters or disorders, identifying key “bridges” (Jones et al., 2021). Strength centrality, adding absolute weights of all connected edges, indicates a node’s influence on network connectivity (Opsahl et al., 2010). These measure identifies bridge symptoms that potentially serve as critical connectors within the network, reflecting their role in the co-occurrence and interaction of depressive and anxiety symptoms.

Network stability and accuracy estimation. To confirm the accuracy and stability of network, we used the bootnet package in R, following the procedures by Epskamp and Fried (2018a,b). In this context, “accuracy” refers to the precision of the estimated edge weights in the network—that is, the extent to which these estimates approximate their true values. We evaluated accuracy by generating 1,000 bootstrap samples to compute 95% confidence intervals (CIs) for the edge weights; narrower CIs indicate higher accuracy. Similarly, “stability” refers to the consistency of the network structure (particularly the centrality indices) when the data is subjected to variations or when subsets of the data are used. We assessed stability through a case-dropping bootstrap method, calculating the CS-coefficient from 1,000 samples. The CS-coefficient (ideally above 0.25) reflects the proportion of the sample that can be dropped while still retaining a correlation of at least 0.7 between the full dataset and the subset estimates of the centrality indices (Epskamp and Fried, 2018a; Epskamp and Fried, 2018b). Lastly, we applied a bootstrap difference test (1,000 samples, alpha = 0.05) to assess network edge and node differences, comparing bootstrapped CIs of edge weights and centrality indices (Epskamp and Fried, 2018a; Epskamp and Fried, 2018b).

#### 2.3.2 Bayesian network analysis

To explore potential causal relationships in the symptom network, we used Directed Acyclic Graph (DAG) to estimate the Bayesian network with the bnlearn package in R 4.2.3 (Scutari, 2009). DAGs help infer potential causal pathways by representing conditional dependencies between variables. We employed a bootstrapped Hill-Climbing (HC) algorithm, following Briganti et al. (2023). The HC algorithm, a score-based method, iteratively modifies edges to maximize a score while keeping the graph acyclic (Russell and Norvig, 2010). Our analysis was strengthened by creating 1,000 bootstrap samples and configuring multiple restarts to avoid local optima, following McNally et al. (2017). After bootstrapping, we synthesized an averaged network from all samples, evaluated using the Bayesian Information Criterion (BIC) for model fit. We also computed arc strengths using the BIC-Gaussian score method to determine the certainty of each directed edge. The final DAG, visualized using the bnlearn package, showed elliptical nodes and edges weighted by arc strengths, highlighting the most reliable causal relationships between symptoms.

#### 2.3.3 Network comparisons between gender

To compare the network structures between different groups, we employed the “Network Comparison Test (NCT)” using the corresponding R package (van Borkulo et al., 2022). NCT enables statistical comparisons of networks using metrics like global strength (total of all absolute edge weights), individual edge weights, and centrality measures such as strength. This allows NCT to identify significant differences in overall network connectivity and the roles/importance of specific nodes and edges. Considering extensive research has consistently shown that females are nearly twice as likely as males to experience depression in their lifetime (Kuehner, 2017), we applied NCT to contrast networks from female and male groups. Thus, we could know whether the observed gender differences extend beyond mere prevalence rates and also reflect differences in the interrelationships among individual symptoms.

## 3 Results

### 3.1 Descriptive statistics

Among 7,471 participants (average age 16.37 ± 0.94), 59.29% were males (*N* = 4,428, average age = 16.39 ± 0.89), and 40.71% were females (*N* = 3,043, average age = 16.35 ± 0.92). The average scores for the PHQ-8 and GAD-7, along with the prevalence of depression and anxiety defined by scores of 10 or higher (Kroenke et al., 2009; Spitzer et al., 2006), are detailed in Table 1. It is noteworthy that the prevalence and total score of depression and anxiety were significantly higher among female participants than male counterparts (*ps* < 0.001). Moreover, females scored significantly higher than males in depressive symptoms and anxiety symptoms, as shown in Supplementary Table 1. The comorbidity rate was 11.31% in the total sample, with no significant difference between genders.

**TABLE 1:** Participant demographics and descriptive statistics of depression and anxiety.

	Mean (S.D.)/(*n*%)		
	Total sample (*N* = 7,421)	Males (*N* = 4,428)	Females (*N* = 3,043)
Age	16.35 (0.92)	16.39 (0.89)	16.35 (0.92)
**Depression: PHQ-8**			
Average total score	5.86 (5.11)	5.17 (4.76)	6.88 (5.42)
Prevalence (scores ≥ 10)	1,551 (20.76%)	595 (19.55%)	812 (26.68%)
**Anxiety: GAD-7**			
Average total score	4.84 (4.53)	4.15 (4.16)	5.84 (4.84)
Prevalence (scores ≥ 10)	1,037 (13.88%)	442 (9.98%)	739 (16.69%)
Comorbidity rate	11.31%	11.53%	11.34%

### 3.2 GGM network analysis of depressive symptoms and anxiety symptoms

**FIGURE 1:**
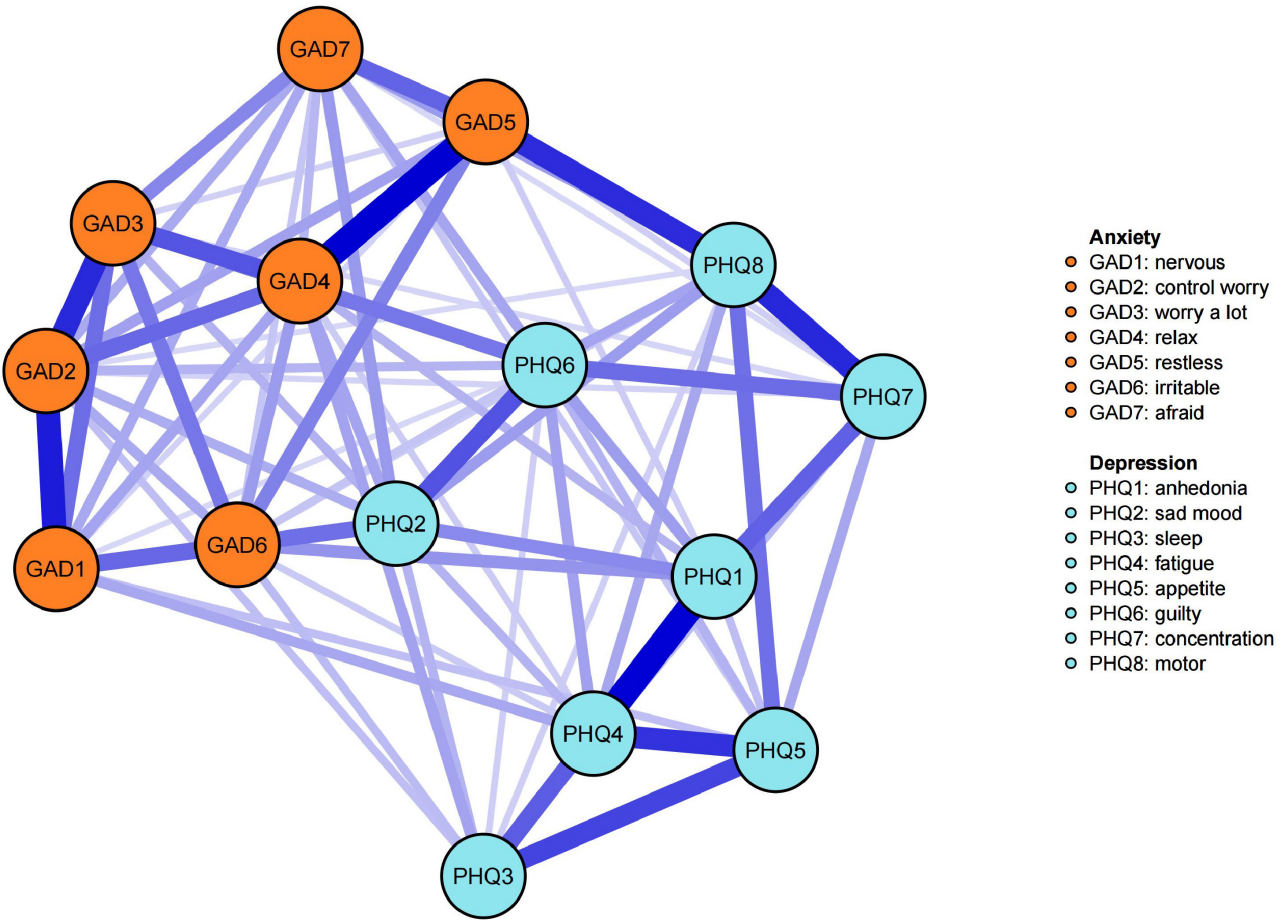
Network structure of depressive and anxiety symptoms. (1) Nodes are color-coded by DSM-5 symptom clusters: orange for anxiety symptoms, blue for depressive symptoms. (2) Edge colors indicate correlations: purple for positive, red for negative, with thickness denoting the strength of pairwise correlations – thicker edges signify stronger correlations.

Network estimation and visualization. Figure 1 displays the GGM network for depressive and anxiety symptoms. Out of 105 potential node pairs, 77 showed associations, leading to a network density of 0.733 and an average edge weight of 0.09. Edge weight details are in the Supplementary Table 2. The network visualization shows clusters of depressive and anxiety symptoms, indicating their inter-connectivity. All network edges were positive, suggesting a positive correlation between these symptoms.

**FIGURE 2:**
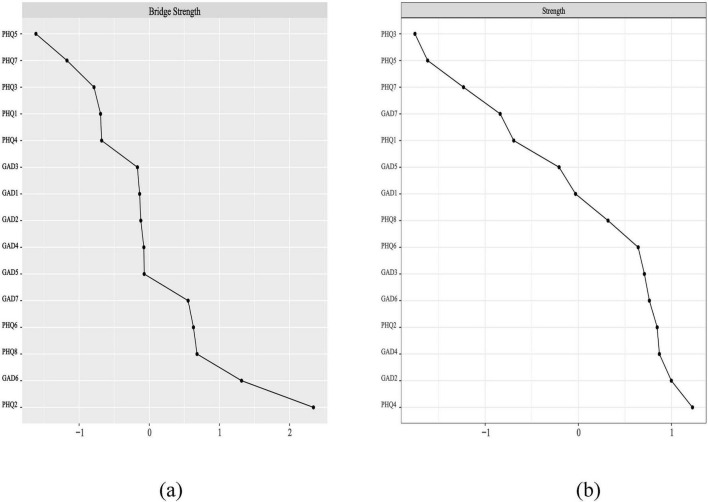
The variables are more evenly distributed across the scale, while in graph **(b)**, they cluster closer to the center. **(a)** Bridge strength (Z-standardized); **(b)** Strength (Z-standardized). GAD1, nervousness; GAD2, loss of control; GAD3, excessive worry; GAD4, trouble relaxing; GAD5, restlessness; GAD6, irritability; GAD7, feeling afraid; PHQ1, anhedonia; PHQ2, sad mood; PHQ3, sleep problems; PHQ4, fatigue; PHQ5, appetite changes; PHQ6, guilt; PHQ7, difficulty concentrating; PHQ8-motor.

Node’s Bridge Centrality and Centrality. Figure 2a shows node bridge strengths, and Supplementary Figure 1 presents results from the non-parametric bootstrap difference test. With the highest bridge strength and significant differences than others (*P* < 0.05, Supplementary Figure 1), sad mood was the bridge symptom of the network. Figure 2b illustrates node strengths, with Fatigue having the highest. Additionally, Supplementary Figure 2’s non-parametric bootstrap difference test indicates that loss of control, trouble relaxing, sad mood, irritability, excessive worry, feeling guilty have significantly higher strengths than other symptoms, these symptoms are thus considered central within the network.

Network stability and accuracy. All results are in Supplementary Figure 3, both the nodes and edges of the network have high stability and accuracy. The results of the Subsetting Bootstrap are shown in Supplementary Figure 3a, and the stability coefficients (CS-Coefficient) of node bridge strength and strength are all 0.75. The results of the edge weight bootstrap procedure are shown in Supplementary Figure 3b. The edge weights in the current sample are the same as the bootstrap sample, which means the network has high stability.

### 3.3 Bayesian network analysis of depressive symptoms and anxiety symptoms

**FIGURE 3:**
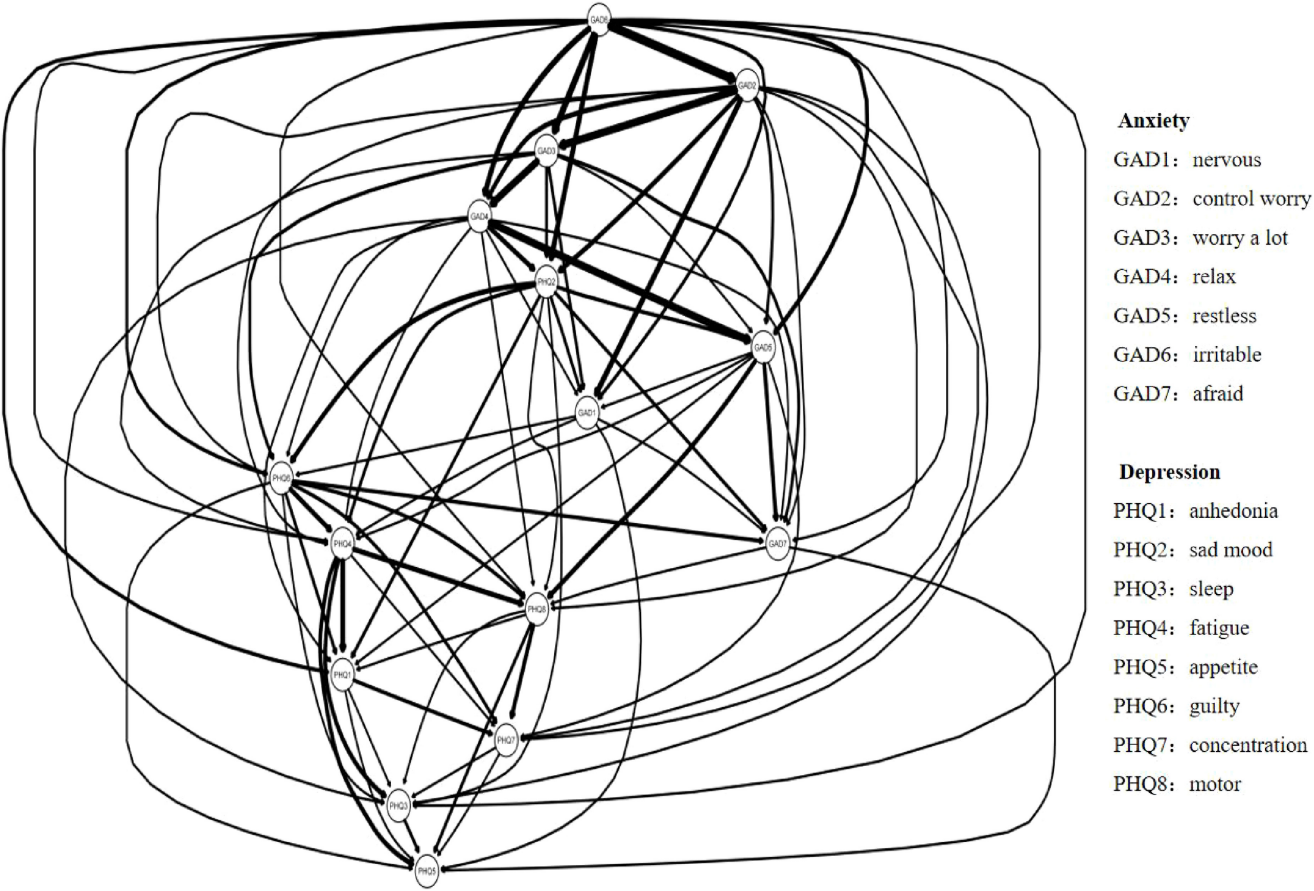
Network diagram illustrating connections between anxiety and depression indicators. Arrows indicate the direction of influence between nodes. Legend lists specific symptoms for each node.

**FIGURE 4:**
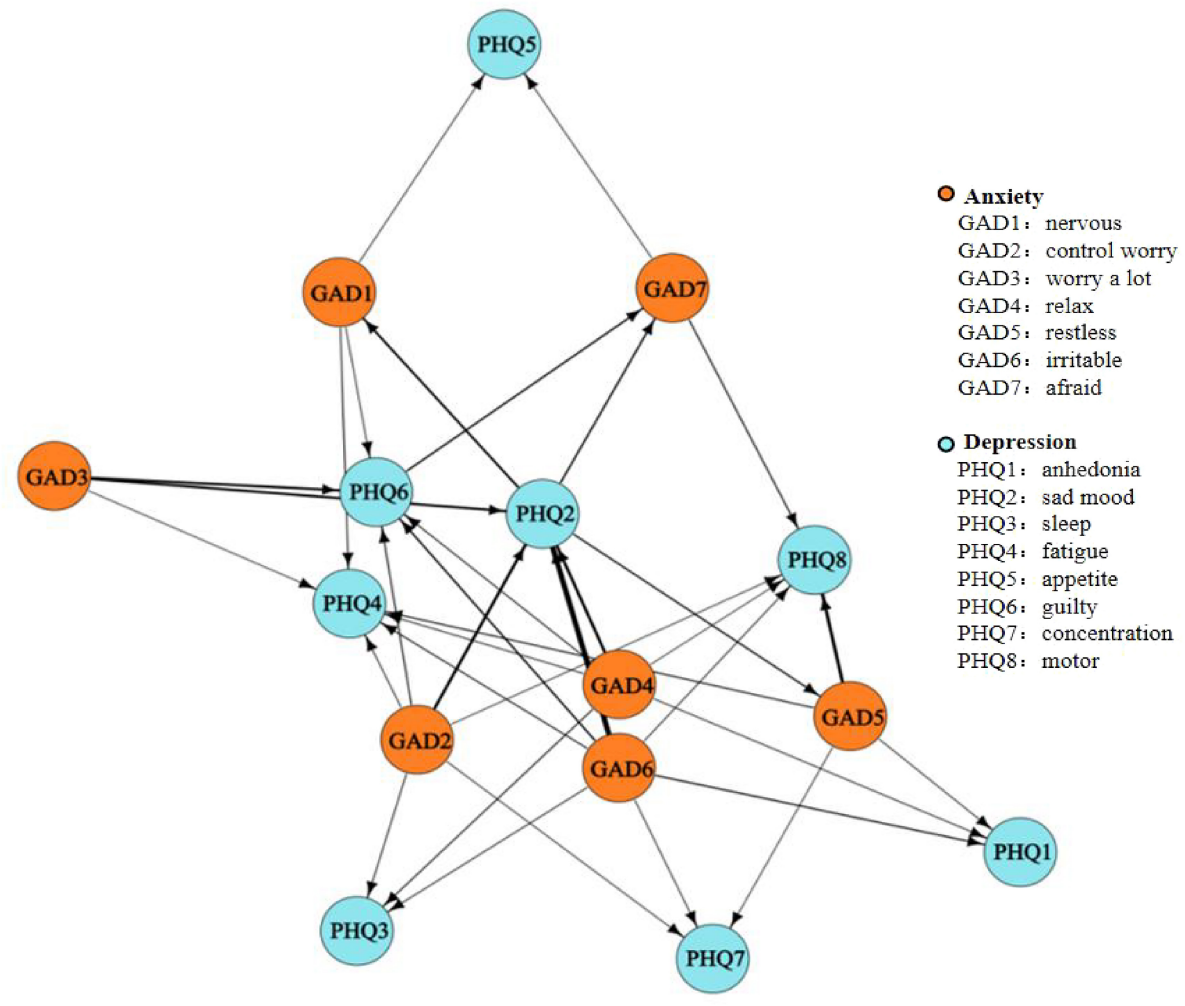
Subgraph of directed edges between depressive and anxiety symptoms. (1) A Subgraph of Directed acyclic graphs (DAG) network nodes represent symptoms and anxiety symptoms and edges represent directed connections between them. (2) The thickness of the arrows represents directional probability.

The DAG analysis results, shown in Figures 3, 4, map potential causal links between depressive and anxiety symptoms. Figure 3 offers an overview of all symptom interactions, and Figure 4 focuses on connections between depressive and anxiety symptoms. In Figure 3, irritability emerges as the initial node, highlighting its potential role in triggering or maintaining other symptoms, thus being a critical target for early intervention. Figure 4 shows a dominant directional flow from anxiety to depressive symptoms, with 31 edges from anxiety to depressive symptoms, suggesting anxiety’s role in developing depressive symptoms. Conversely, the transition from depressive to anxiety symptoms is less evident, with only four edges, mainly from sad mood and one from guilty. This asymmetry indicates that while depressive symptoms can lead to anxiety, this is less common than the reverse.

### 3.4 Network comparison test between gender

The networks showed notable gender differences in global strength, with females’ network exhibiting a higher global strength (7.04) than the males’ network (6.90; *P* < 0.01), which suggests a density connections of symptoms among females (Borsboom and Cramer, 2013; Cramer et al., 2010). Specifically, the symptom of anhedonia presented a significantly higher strength in the females’ network than males’ network (diff_PHQ1 = −0.17, *P* < 0.05), pointing to its greater relevance or impact among females.

## 4 Discussion

As the first study to apply network analysis specifically to depressive and anxiety symptoms among Chinese vocational high school students, this research systematically identified critical nodes within both directed (*Bayesian-directed acyclic graph, DAG*) and undirected (*Gaussian graphical model, GGM*) symptom networks. Our approach revealed one bridge symptom and seven core symptoms, explored potential directional relationships between depressive and anxiety symptoms, and examined gender differences in both prevalence and network structure. Furthermore, we confirmed the stability and accuracy of our network models, ensuring robust conclusions. We found one bridge symptom and seven core symptoms, uncovered bidirectional associations between symptoms and gender differences in GGM network structure. These findings provide novel insights from a network perspective. To validate these exploratory findings, future studies should prioritize longitudinal designs to track symptom dynamics and experimental interventions targeting identified core/bridge symptoms.

A key finding of our study is the mutual influence between depressive and anxiety symptoms, with a more pronounced trend from anxiety symptoms to depressive symptoms. This pattern suggests that anxiety may be an earlier and more influential factor in the development of comorbidity. It aligns with existing literature indicating that anxiety can predict depression, with the predictive effect of anxiety on depression being stronger in the short term and influenced by the baseline level of anxiety (Jacobson and Newman, 2017), potentially acting as a catalyst for the development of depressive symptoms. Our study conducted following a new COVID-19 outbreaks, students faced the uncertainty and threat brought about by the pandemic may lead to increased anxiety symptoms in individuals (Bendau et al., 2021), and the absence of effective coping strategies lead to an exacerbation of anxiety and depressive symptoms (Miola et al., 2023). Thus, the development and implementation of positive coping strategies are crucial in mitigating the severity of these symptoms and promoting mental health resilience.

Notably, irritability emerged as the initial node in the DAG network, influencing all other symptoms and highlighting its importance for preventive interventions. Irritability also emerged as a core symptom in Chinese female nursing students and a bridge symptom in epilepsy patients (He et al., 2023; Ren et al., 2021). A recent meta-analysis of 121 studies confirmed irritability as a transdiagnostic status across youth mental-health disorders (Kim and Asbury, 2020). Cross-lagged-panel work further indicates that elevations in irritability at Time 1 precipitate increases in worry and somatic tension six months later, but not vice-versa (Chin et al., 2025). Considering our research carried out followed a new COVID-19 outbreaks, with a significant reductions in social activities (Losiewicz et al., 2023), and heightened perceptions of risk (Webb, 2021). These threat factors, uncertainty, and isolation during COVID-19 pandemics, have been consistently associated with heightened stress responses and the emergence of anxiety symptoms (Buheji et al., 2020). Because our DAG implies top-down influence, targeting irritability may yield a cascade effect. Irritability should therefore be prioritized for early identification and intervention to prevent further mental health issues in vocational high school students. Youth-specific RCT already showed medium-to-large reductions in global impairment after irritability-focused program—an exposure-based CBT protocol. Adapting these brief (6–8 session) formats to homeroom periods could provide cost-effective, population-level prevention. We recommend incorporating a single-item irritability screener into routine school health checks and intervention in frustration-tolerance skills (Naim et al., 2024).

Importantly, sad mood exerts a significant influence on symptoms transition across depression and anxiety. Identified as a bridge symptom in the GGM network, consistent with various studies (He et al., 2023; Ren et al., 2021; Wang et al., 2020), and was found to demonstrate potential directional relationships with both anxiety and depression symptoms in the DAG network for the first time. This bi-directionality indicates a complex interplay where sad mood not only results from but also contributes to the exacerbation of other symptoms within the network. Our results corroborate Clark and Watson’s (1991) tripartite model of anxiety and depression, which posits negative affectivity, like sad mood and despair, as a shared characteristic of both conditions. The significant role of these symptoms in cross-disorder symptom transmission becomes evident due to their shared nature. Building on Kaiser et al. (2021) recommendation for targeted interventions on bridge symptoms, and giving the stronger evidence supporting the role of sad mood in the network, we advocate for therapeutic interventions focused on this symptom to potentially reduce the occurrence and progression of comorbid depression and anxiety.

Moreover, fatigue, loss of control, trouble relaxing, sad mood, irritability, excessive worry, and guilt were the core symptoms. Notably, some researchers found similar core symptoms (e.g., low energy and trouble relaxing) in university students, but with physical activity and stress management as key protective factors—a contrast highlighting the need for age-specific interventions (Sun et al., 2024). Regarding depressive symptoms, while fatigue and sad mood are well-recognized core symptoms (Beard et al., 2016; He et al., 2023; Kaiser et al., 2021), the prominence of guilt is a novel finding, possibly reflecting the cultural and educational context in East Asian Confucian societies, where academic failure often leads to feelings of inadequacy or guilt (Fwu et al., 2017). Regarding anxiety symptoms, their identification as core may be attributed to the timing of our study during the COVID-19 pandemic. Another network analysis conducted during the COVID-19 pandemic also highlighted the central role of anxiety symptoms in comorbid presentations. Loss of control was also a core symptom in nursing students during the COVID-19 (Bai et al., 2021), irritability and trouble relaxing showed high centrality during the after peak stage of Covid-19 pandemic (Naim et al., 2024). These findings emphasize the significant impact of anxiety symptoms on mental health during the pandemic and suggest targeting these symptoms for prevention and intervention in similar public health crises.

Finally, the study found significant gender differences in the prevalence of depression and anxiety, network global strength, and the strength of anhedonia, all of which were higher in females. This suggests more severe symptoms and a more connected network in the female. Such gender disparity aligns with literature indicating females experience more severe depression and anxiety challenges (Bang et al., 2020). The emergence of this gender difference is consistent with Sun et al. (2024)’s findings in primary/middle school students, in which screen time had stronger associations with negative emotions in females. This suggests that gender-specific vulnerability patterns may persist across developmental stages, though the contributing factors may differ. The stronger symptom connection network may be one of the possible reasons to explain why the depression and anxiety prevalence in females is higher than in males. According to Borsboom (2017), in tightly connected networks, symptoms can sustain activity even after initial triggers subside, leading to disorder states. Thus, females, with a denser network, are more susceptible susceptible to comorbid depression and anxiety. Additionally, anhedonia showed significantly higher strength in females, but literature on its gender-specific impact is mixed (Bolton et al., 2018; Chan et al., 2012), indicating a need for further research. In general, our study not only identifies a higher prevalence of comorbid depression and anxiety in females but also, from a network analysis perspective, sheds light on the potential underlying mechanisms.

This study utilized network analysis to examine depression and anxiety symptom patterns and gender differences in vocational high school students. In doing so, we have provided insights into symptom-level understanding of depression and anxiety in this large but overlooked target group, and provided recommendations for subsequent prevention and intervention. We discovered potential directional relationships in the spread of symptoms across diseases, as well as key symptoms within the network structure. Based on these findings and relevant theories, we proposed directions for future interpretation and practical application based on relevant theories and our findings, and verified the existence of gender differences, providing an explanation from a network perspective. Firstly, we recognized anxiety symptoms, especially irritability as early indicators for comorbidity onset and progression, advocating for prompt preventive measures. Concurrently, we suggested therapeutic interventions focused on sad mood to effectively prevent further development and exacerbation of comorbid depression and anxiety. Lastly, considering the higher prevalence and susceptibility in females, we recommend prioritizing mental health resources for female students in settings with limited resources.

The network analysis and sample used in this study to reveal patterns of depression and anxiety symptoms have several limitations. First, the study’s cross-sectional nature means the DAG network only suggests potential directional relationships. Time-series or longitudinal studies are needed for deeper insights into symptom activation spread and symptom causality (Forbes et al., 2017). Second, only DSM-5 symptoms of anxiety and depression were included. Non-DSM-5 symptoms could also be crucial in these networks (Fried et al., 2016). This may limit the comprehensiveness of the identified symptom interactions. Future studies could expand symptom inventories to capture broader clinical profiles. Future research should consider a broader range of symptoms to more accurately explain comorbidity. Third, the present study is limited to first-year vocational high school students in China, without comparing their networks to those of students from other grades or regular high schools, which limits the understanding of both commonalities and differences across different student groups. Additionally, the regional focus on Chongqing may constrain the generalizability of findings to other socioeconomic or cultural contexts in China. Future studies could expand to multi-grade cohorts and diverse school types to validate these patterns. Network connectivity differences may exist across different samples or populations (Schultz et al., 2019). Fourth, while our study provides novel network insights into depression and anxiety among vocational students, we did not assess developmental factors such as identity formation or hormonal changes that may influence symptom networks across different adolescent populations (Sun et al., 2024; Sun et al., 2025). Fifth, approximately 16% of responses were ultimately excluded—primarily due to failed validity checks or incomplete data. These exclusions were necessary to ensure the reliability of the network analysis; however, we acknowledge that students experiencing greater psychological distress (e.g., severe anxiety or depression) may have been more likely to provide invalid or incomplete responses. Further research comparing these groups could lead to more tailored interventions. Including clinical samples in future studies could also help verify or challenge these findings.

Despite its limitations, the present study yields critical insights into the depression and anxiety among secondary vocational school students. These implications are crucial for both educational policy and mental health intervention strategies. Firstly, early psychological assessment is crucial, with irritability and sad mood as key indicators. Systematic screening for these symptoms in schools can enable early intervention, potentially altering comorbidity trajectories. Secondly, the pronounced gender disparity in comorbidity, particularly among female students, underscores the need for gender-specific psychological support strategies, such as tailored support groups, counseling, and psycho-educational programs. However, future research should prioritize elucidating the mechanisms underlying this disparity rather than solely detecting it. Potential contributors warrant systematic investigation. Longitudinal designs integrating multi-modal data could disentangle whether observed network density differences reflect biological predispositions, contextual stressors, or their interaction. Such mechanistic insights are critical for developing interventions that address root causes rather than symptomatic manifestations of the gender gap. Third, our findings are consistent with recent studies demonstrating that Chinese students continued to experience significant mental health challenges even after the relaxation of COVID-19 restrictions (Cai et al., 2025). These persistent effects, particularly among secondary school students, suggest that the psychological impacts of the pandemic have persisted long after the acute crisis period, reinforcing the continued relevance of our findings in the post-pandemic educational context. Lastly, integrating mental health literacy into the vocational curriculum, with a focus on psychological resilience and access to support, is essential. This approach aims to foster a mentally resilient and informed student body.

## 5 Conclusion

The network analysis identified sad mood (PHQ2) as the primary bridge symptom linking depression and anxiety, while fatigue (PHQ4) and six other symptoms (loss of control, trouble relaxing, irritability, excessive worry, guilt, and difficulty concentrating) emerged as core nodes with high centrality. Directed acyclic graph analysis further revealed that anxiety symptoms (e.g., irritability) predominantly activated depressive symptoms, with irritability (GAD6) serving as the initial trigger in the symptom cascade. Notably, female participants exhibited stronger global network connectivity, suggesting denser symptom interactions that may underlie their higher prevalence of comorbid depression and anxiety. These findings underscore the urgency of gender-sensitive interventions targeting bridge and core symptoms—particularly irritability and sad mood—to disrupt symptom propagation pathways in this vulnerable population.

## Data Availability

Publicly available datasets were analyzed in this study. This data can be found here: if data sets are required, contact the corresponding author.
