# Supplementary material for: Exploring the co-occurrence of depression and anxiety symptoms among Chinese vocational high school adolescents through a network approach
Source: Front Psychol. 2025 Aug 11;16:1572011. doi: 10.3389/fpsyg.2025.1572011 (PMC12376899; doi:10.3389/fpsyg.2025.1572011)
Supplement: Supplementary file 1 [file Table_1.docx]

Table S1 Strength of each symptoms in network of depressive symptoms

| symptoms | PHQ1 | PHQ2 | PHQ3 | PHQ4 | PHQ5 | PHQ6 | PHQ7 | PHQ8 |
| --- | --- | --- | --- | --- | --- | --- | --- | --- |
| Strength | 0.813 | 0.832 | 0.685 | 0.985 | 0.741 | 0.912 | 0.748 | 0.819 |

Table S2 Strength and Bridge Strength of each variable in network of both depressive symptoms and stressful life events

| variables | Strength | bridge Strength |  | variables | Strength | bridge Strength |  | variables | Strength | bridge Strength |
| --- | --- | --- | --- | --- | --- | --- | --- | --- | --- | --- |
| PHQ1 | -0.802 | -0.036 |  | ALSEC5 | -0.413 | 1.205 |  | ALSEC17 | 1.046 | 0.620 |
| PHQ2 | -0.529 | 0.252 |  | ALSEC6 | 0.782 | 2.950 |  | ALSEC18 | 2.607 | -0.714 |
| PHQ3 | -1.696 | -0.724 |  | ALSEC7 | -0.269 | 0.476 |  | ALSEC19 | -0.596 | -0.981 |
| PHQ4 | -0.432 | -0.481 |  | ALSEC8 | -0.549 | 0.688 |  | ALSEC20 | 0.102 | -1.382 |
| PHQ5 | -1.175 | -0.487 |  | ALSEC9 | 1.044 | 1.612 |  | ALSEC21 | 1.424 | -0.905 |
| PHQ6 | 0.069 | 0.309 |  | ALSEC10 | -0.427 | 0.133 |  | ALSEC22 | 0.804 | 0.527 |
| PHQ7 | -1.353 | 0.102 |  | ALSEC11 | 0.778 | -0.618 |  | ALSEC23 | 1.081 | -0.984 |
| PHQ8 | -1.054 | 0.121 |  | ALSEC12 | -0.056 | -1.049 |  | ALSEC24 | -0.332 | -0.209 |
| ALSEC1 | 0.794 | 1.036 |  | ALSEC13 | 0.145 | -1.129 |  | ALSEC25 | 1.691 | 0.413 |
| ALSEC2 | -0.465 | 0.221 |  | ALSEC14 | -0.957 | -1.164 |  | ALSEC26 | 0.762 | -1.248 |
| ALSEC3 | 0.355 | 1.552 |  | ALSEC15 | 0.462 | -0.622 |  | ALSEC27 | -2.091 | -0.907 |
| ALSEC4 | -0.039 | 1.682 |  | ALSEC16 | -0.712 | -0.259 |  |  |  |  |


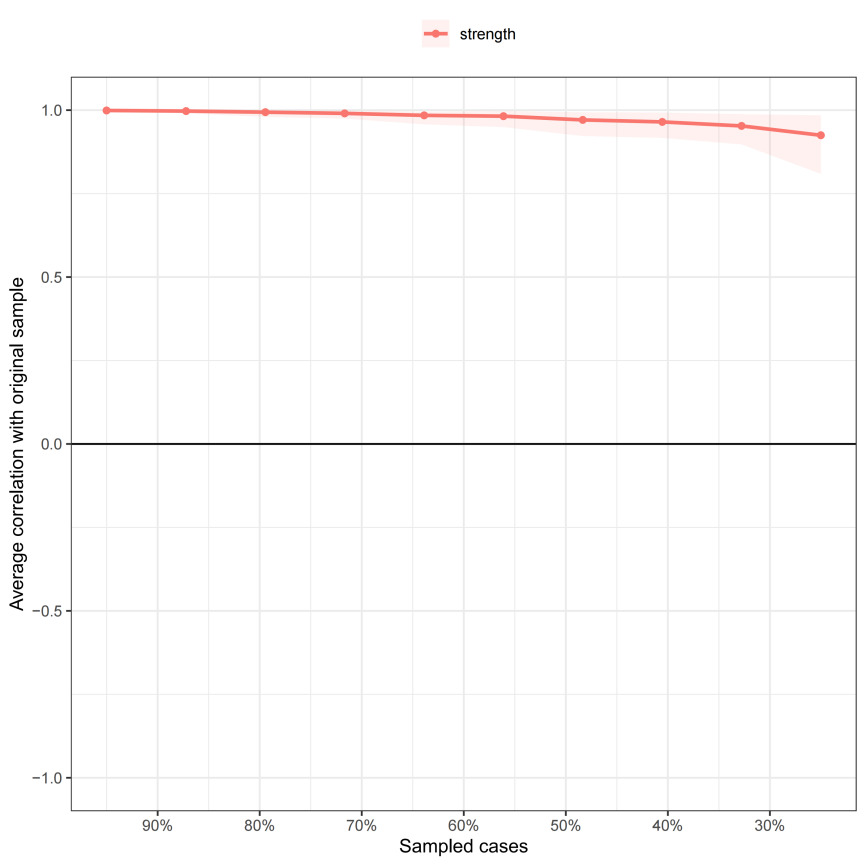


Figure S1.a. Centrality Stability of depressive symptoms network structures.

*
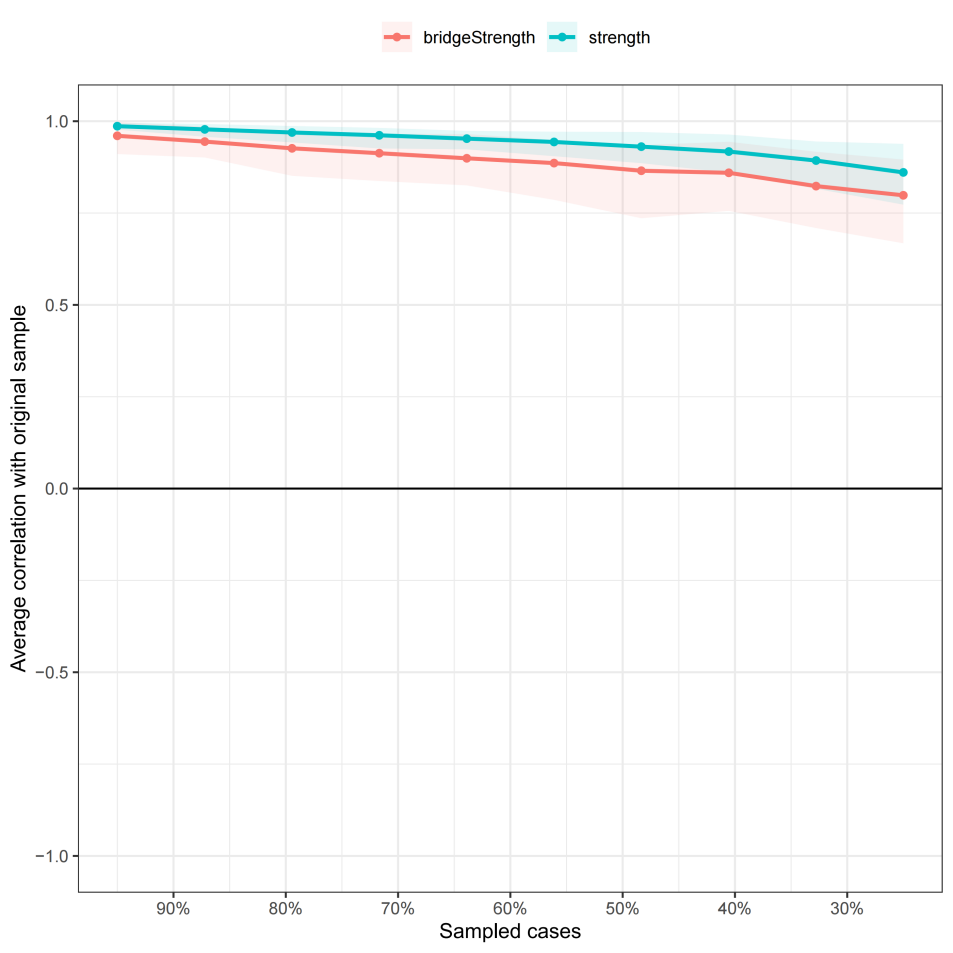
*

Figure S1.b Centrality Stability of depressive symptoms and ALSEC items’ network structures.

*Note. Figure S1. Centrality Stability of network structures. The x-axle indicates the included portion of cases, and the y-axle indicates the correlations between the original centrality indices with the estimated centrality after dropping part of the cases. Lines with different colors represent different network properties. The shades indicate the range from the 2.5th quantile to the 97.5th quantile.*


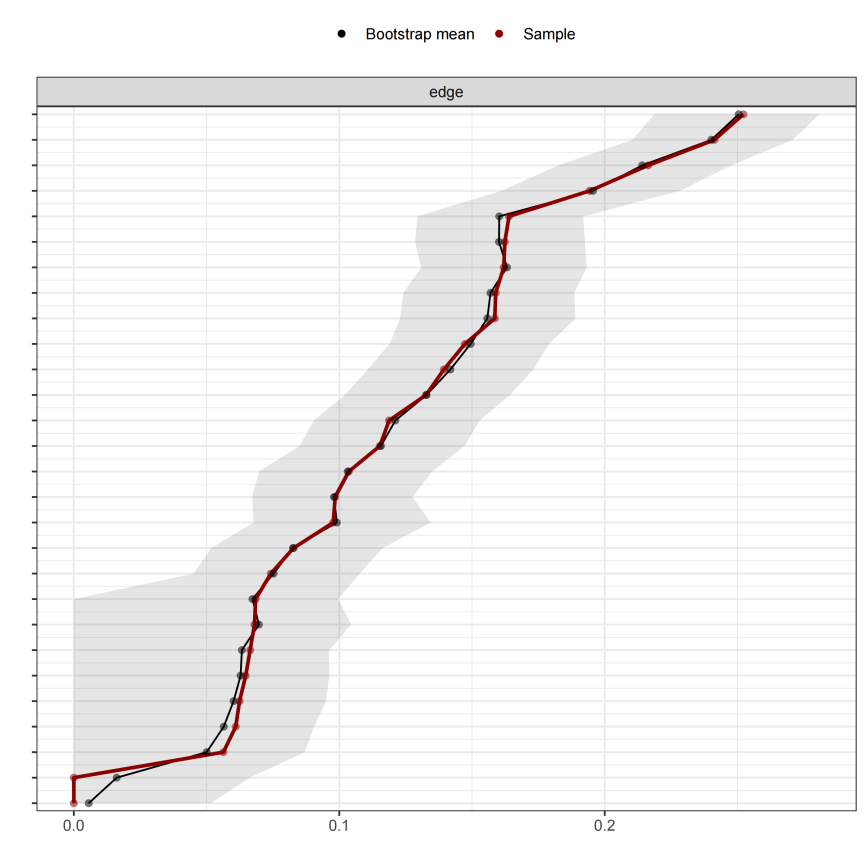


*Figure S2.a. Stability of depressive symptoms’ Edge Weights*

*
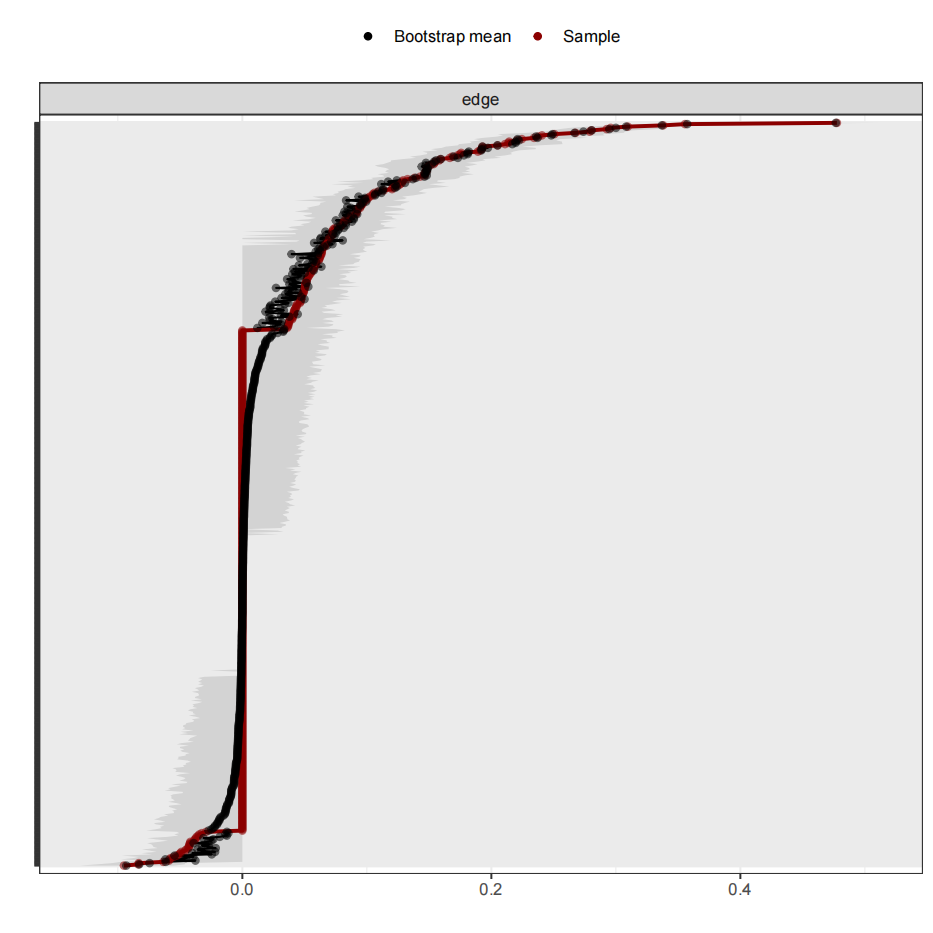
*

*Figure S2.b. Stability of depressive symptoms and ALSEC items’ Edge Weights*

*Note. Figure S2. Nonparametric bootstrapped difference test for Stability of Edge Weights. The x-axis indicates the edge weights and the y-axis indicates the nodes linked by the edges. The black dots denote the mean value of the bootstrapped edge weights and the red dots denote the edge weights from current sample. The black lines denote the 95% confidence intervals of the bootstrapped sample.*


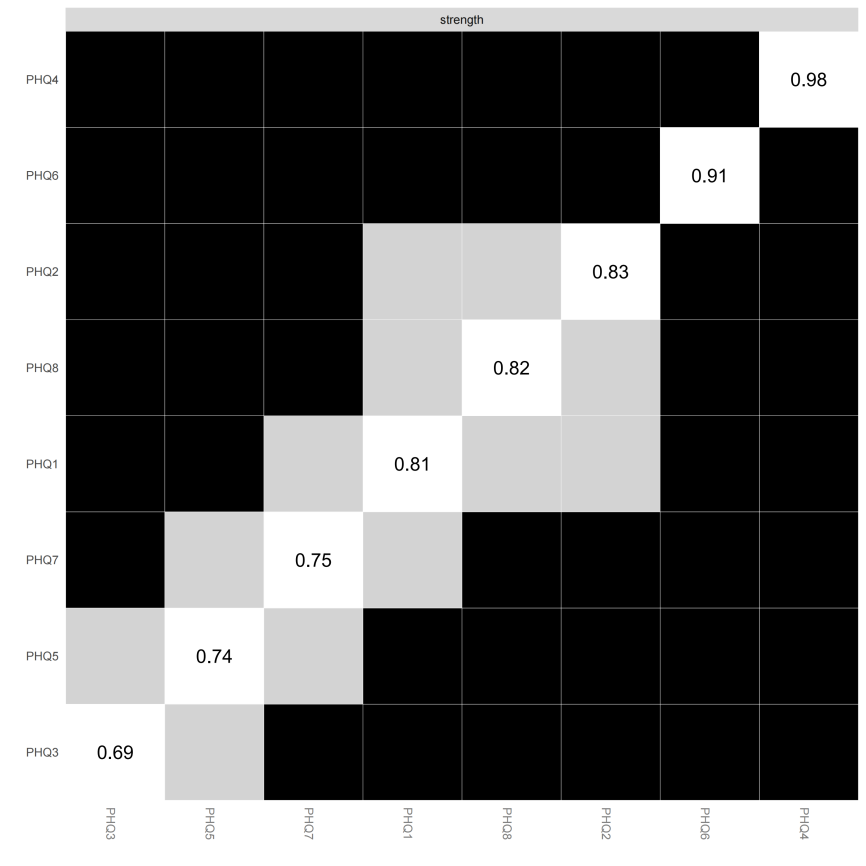


*Figure S3. Nonparametric bootstrapped difference test for strength.*

*Note. Figure S3. Nonparametric bootstrapped difference test for strength. The matrix displays the results of nonparametric bootstrapped difference tests of strength and bridge strength between each symptoms and items. Each cell represents the bootstrapped estimate of the difference between the symptoms and items as indicated by the corresponding row and column headers. Gray boxes indicate no difference between nodes, whereas black boxes indicate significant difference (α = 0.05). Values reported in the diagonal represent the strength values of each node.*

*
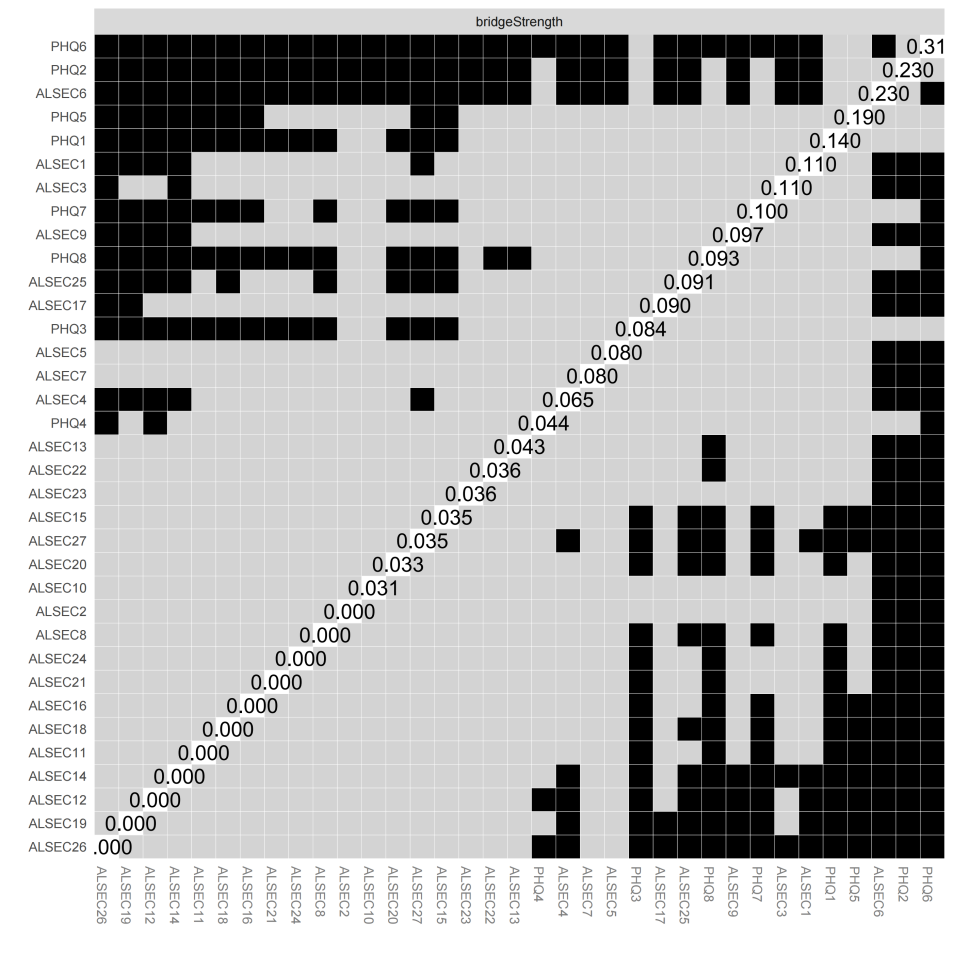
*

*Figure S4. Nonparametric bootstrapped difference test for bridge strength.*


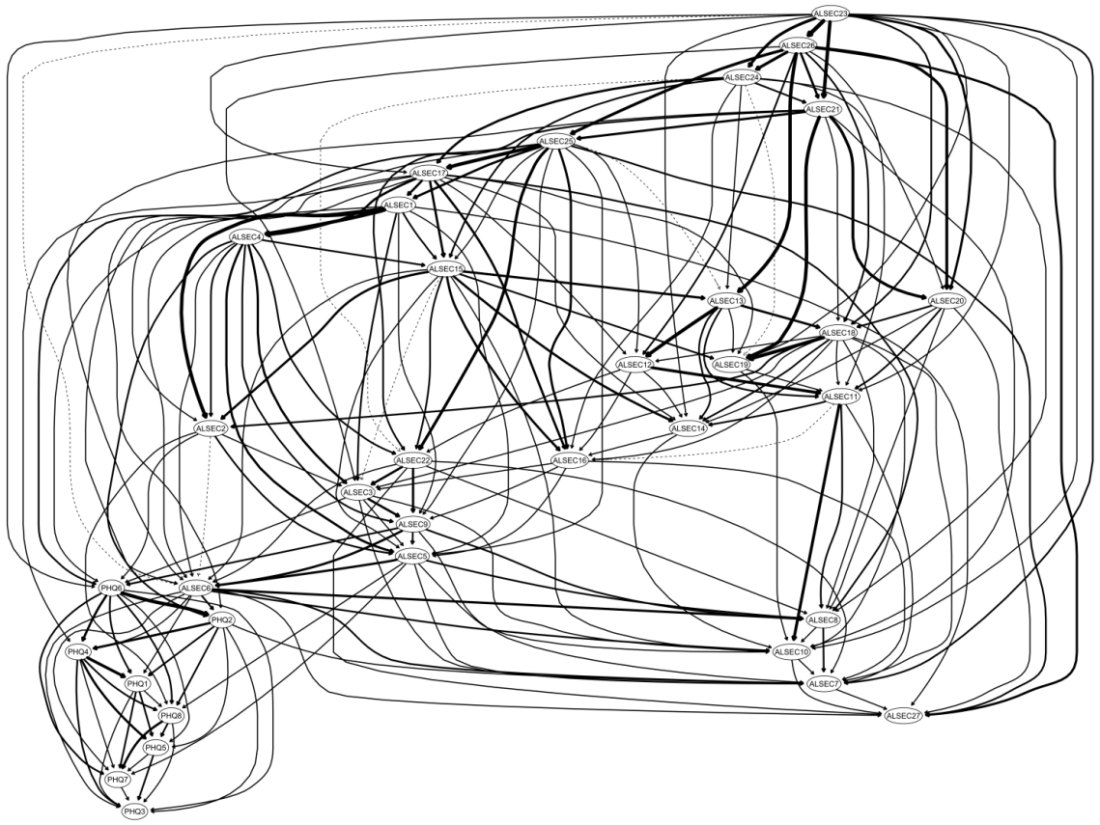


*Figure S5. DAG network of depression symptoms and stressful life events*
